# Supplementary material for: SP140–RESIST pathway regulates interferon mRNA stability and antiviral immunity
Source: Nature. 2025 Jun 11;643(8074):1372–80. doi: 10.1038/s41586-025-09152-2 (PMC12310523; doi:10.1038/s41586-025-09152-2)
Supplement: Supplementary file 1 — Raw gels and immunoblots for all figures with cropping indicated by red boxes. [file 41586_2025_9152_MOESM1_ESM.pdf]

nature portfolio

https://doi.org/10.1038/s41586-025-09152-2

Supplementary information

SP140–RESIST pathway regulates interferon mRNA stability and antiviral immunity

In the format provided by the authors and unedited

Figure 3a

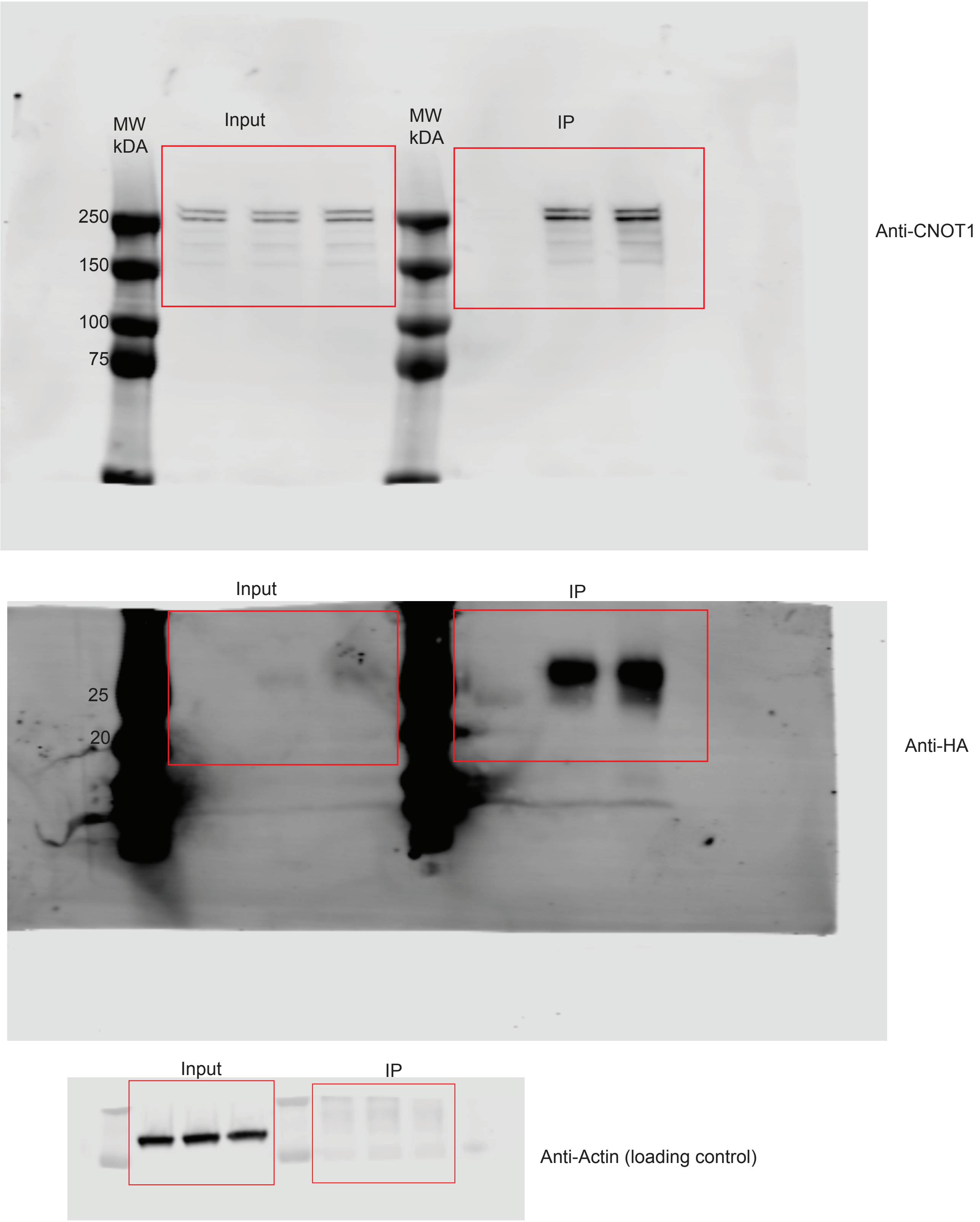

Supplementary Information Figure 1. Full scans of immunoblots.

Figure 4e

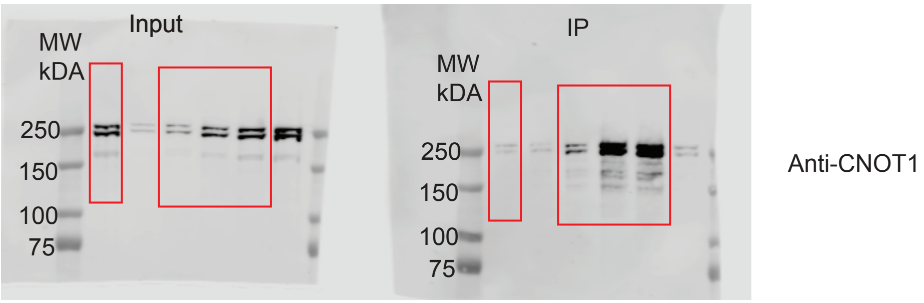

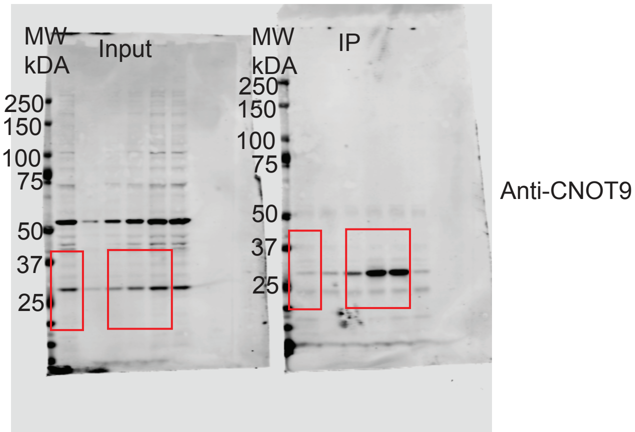

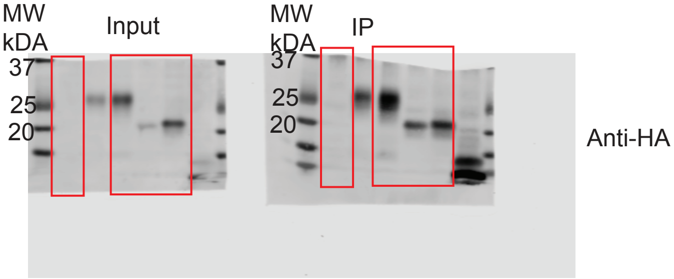

Supplementary Information Figure 2. Full scans of immunoblots and gels.

Figure 4g

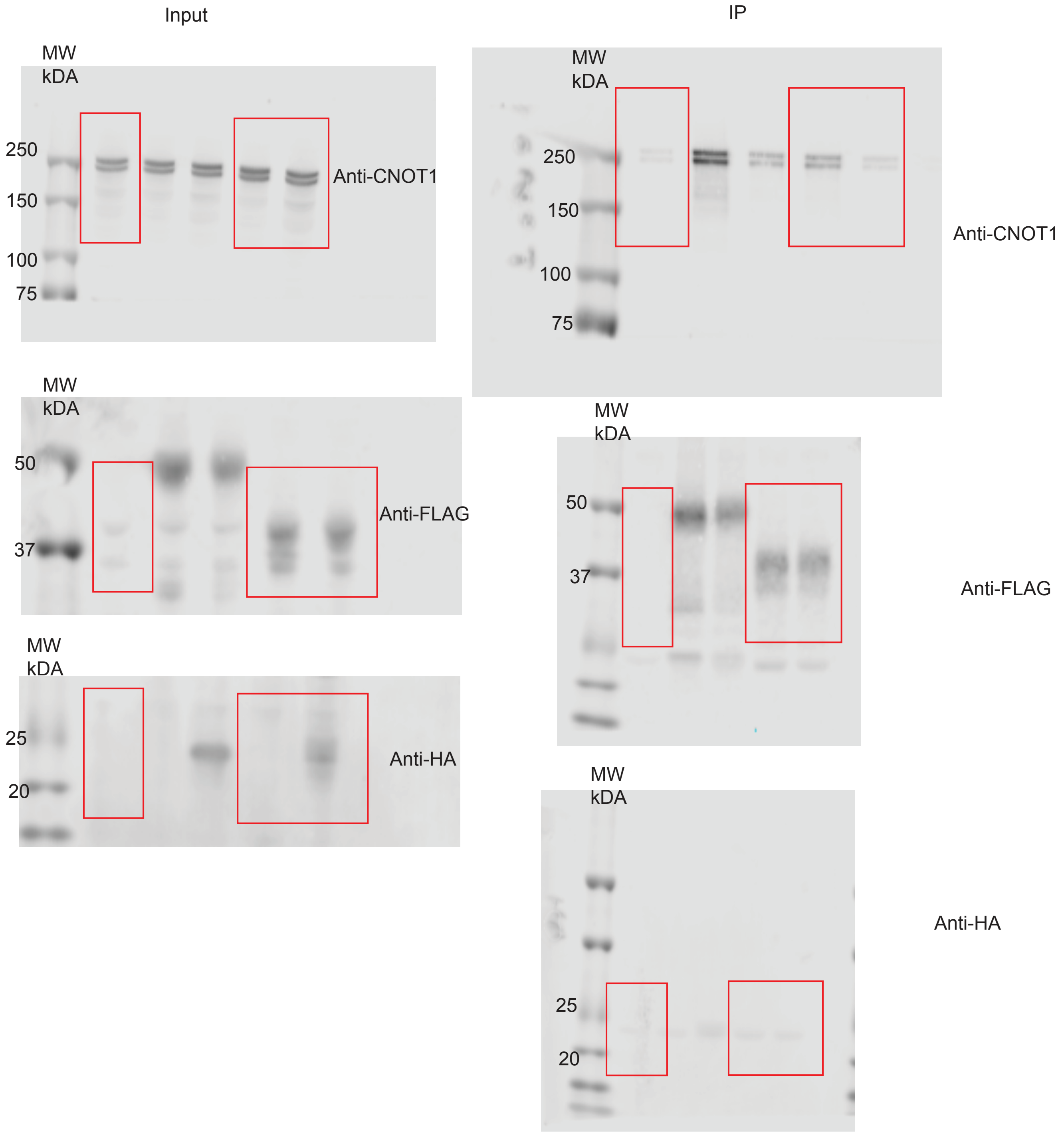

Supplementary Information Figure 3. Full scans of immunoblots.

Extended Data Figure 3c

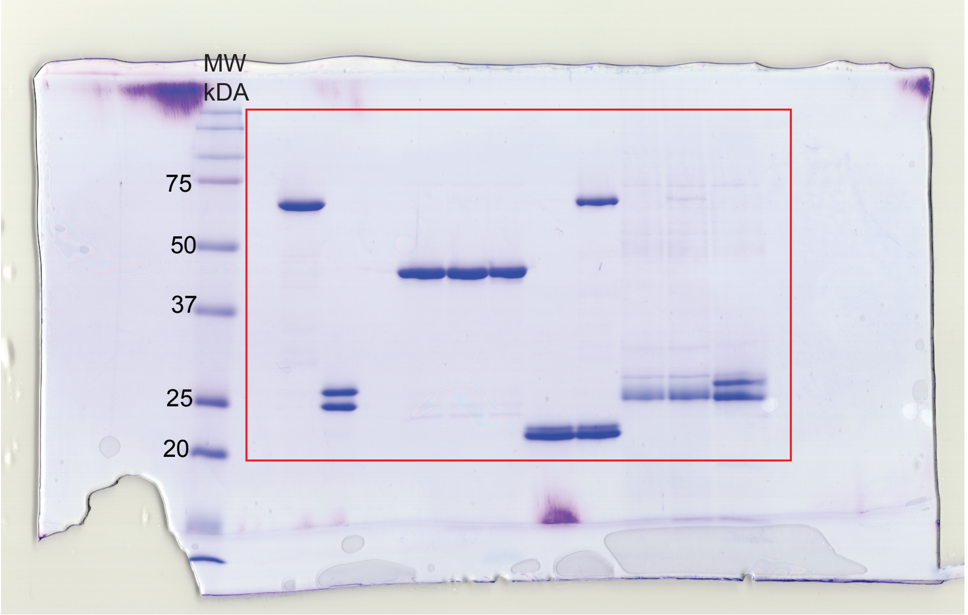

Figure 4c

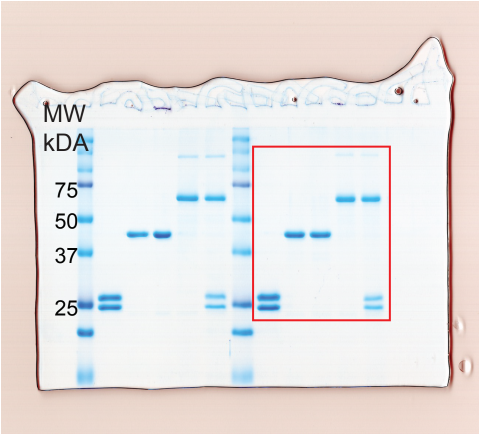

Extended Data Figure 8

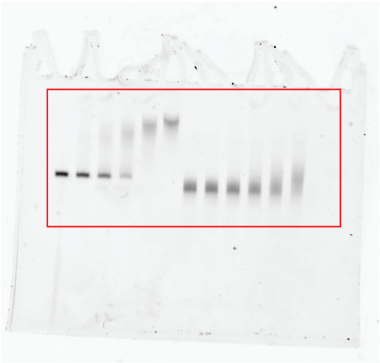

Supplementary Information Figure 4. Full scans of gels.

Extended Data Figure 7b

Left

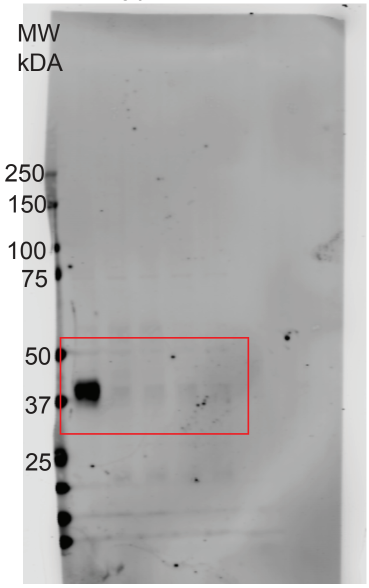

Anti-TTP

Right

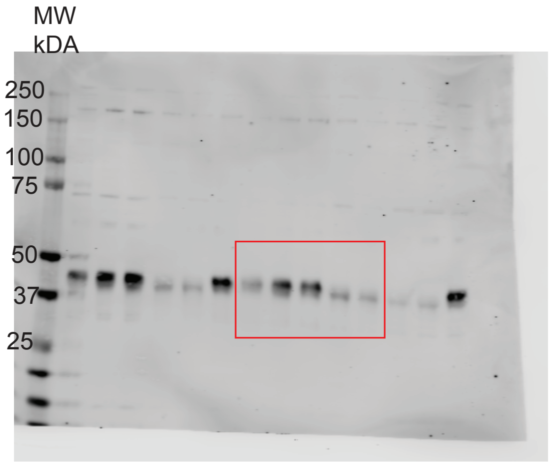

Anti-ZFP36L1

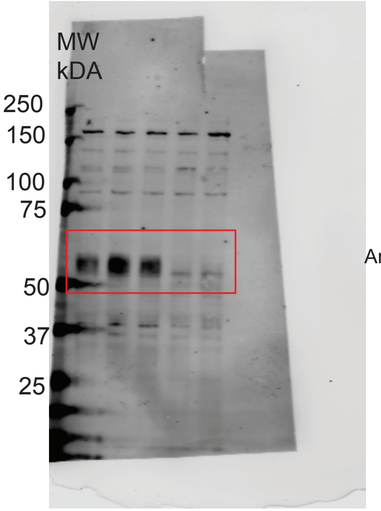

Anti-ZFP36L2

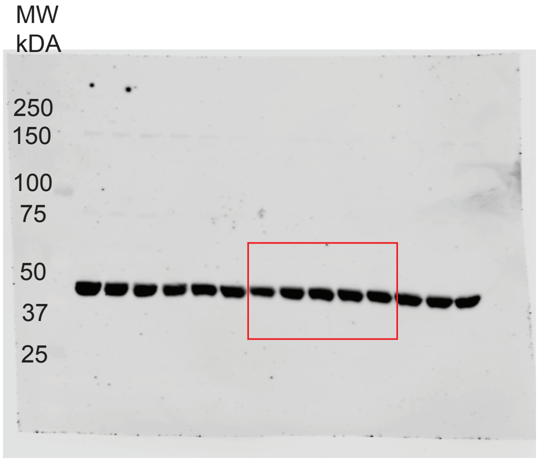

Anti-ACTIN for anti
-ZFP36L1
(loading control)

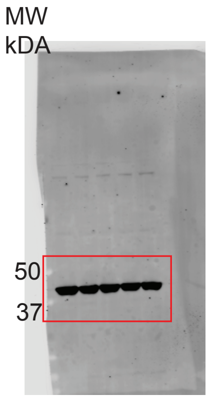

Anti-ACTIN for anti-ZFP36L2 and anti-TTP
(loading control)

Supplementary Information Figure 5. Full scans of immunoblots.

Extended Data Figure 7a

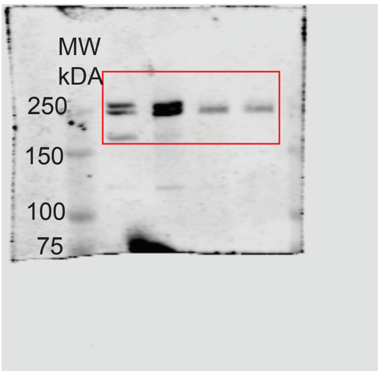

Anti-CNOT1

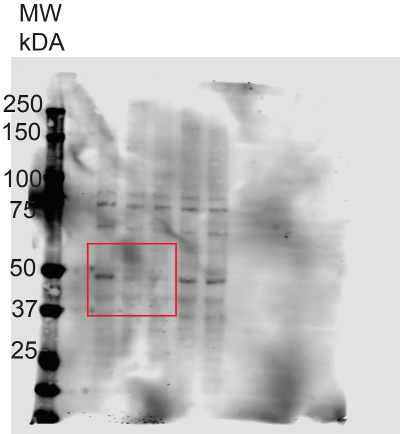

Anti-CNOT11

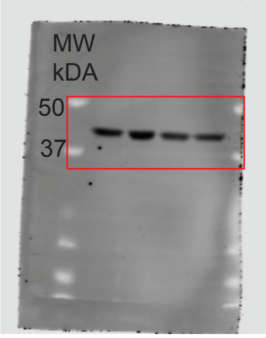

Anti-ACTIN for CNOT1
(loading control)

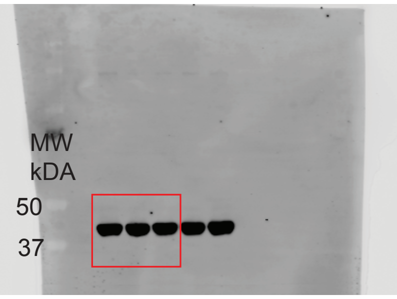

Anti-ACTIN for CNOT11
(loading control)

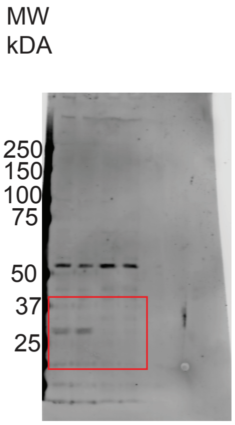

Anti-CNOT9

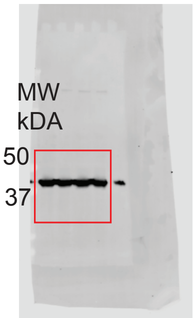

Anti-ACTIN for CNOT9
(loading control)

Supplementary Information Figure 6. Full scans of immunoblots.

Extended Data Figure 9

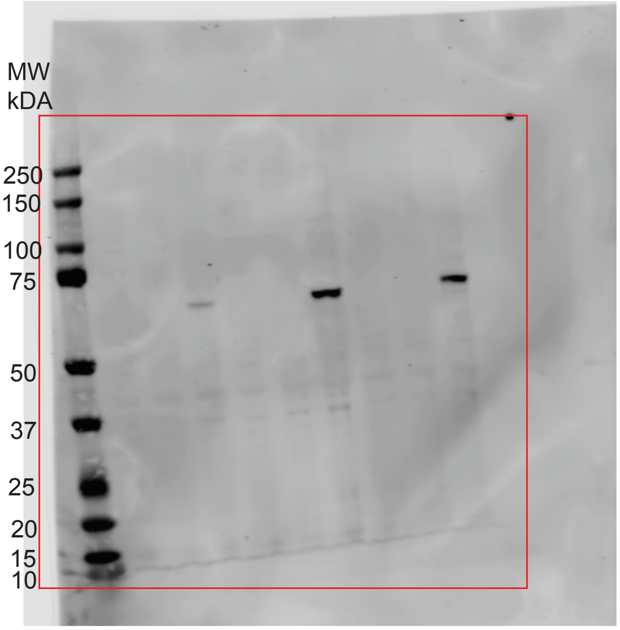

Anti-HA

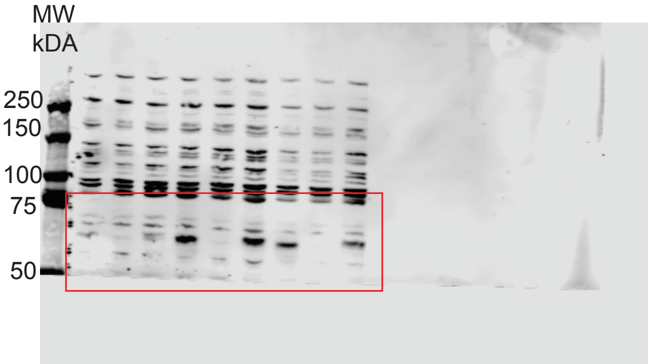

Anti-SP140

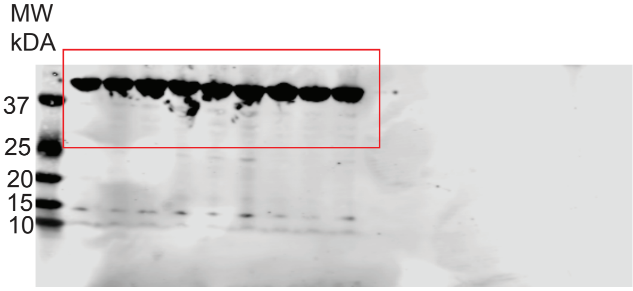

Anti-ACTIN (loading control for anti-SP140 blot, sample processing gel for anti-HA blot).

Supplementary Information Figure 7. Full scans of immunoblots.
