# Supplementary material for: SP140–RESIST pathway regulates interferon mRNA stability and antiviral immunity
Source: Nature. 2025 Jun 11;643(8074):1372–80. doi: 10.1038/s41586-025-09152-2 (PMC12310523; doi:10.1038/s41586-025-09152-2)
Supplement: Supplementary file 2 — Reporting Summary [file 41586_2025_9152_MOESM2_ESM.pdf]

nature portfolio

Corresponding author(s): Russell E. Vance
Last updated by author(s): Mar 29, 2025

Reporting Summary

Nature Portfolio wishes to improve the reproducibility of the work that we publish. This form provides structure for consistency and transparency in reporting. For further information on Nature Portfolio policies, see our Editorial Policies and the Editorial Policy Checklist.

Statistics

For all statistical analyses, confirm that the following items are present in the figure legend, table legend, main text, or Methods section.

n/a	Confirmed	
☐	☒	The exact sample size (n) for each experimental group/condition, given as a discrete number and unit of measurement
☐	☒	A statement on whether measurements were taken from distinct samples or whether the same sample was measured repeatedly
☐	☒	The statistical test(s) used AND whether they are one- or two-sided<br>*Only common tests should be described solely by name; describe more complex techniques in the Methods section.*
☒	☐	A description of all covariates tested
☐	☒	A description of any assumptions or corrections, such as tests of normality and adjustment for multiple comparisons
☐	☒	A full description of the statistical parameters including central tendency (e.g. means) or other basic estimates (e.g. regression coefficient) AND variation (e.g. standard deviation) or associated estimates of uncertainty (e.g. confidence intervals)
☐	☒	For null hypothesis testing, the test statistic (e.g. F, t, r) with confidence intervals, effect sizes, degrees of freedom and P value noted<br>*Give P values as exact values whenever suitable.*
☒	☐	For Bayesian analysis, information on the choice of priors and Markov chain Monte Carlo settings
☒	☐	For hierarchical and complex designs, identification of the appropriate level for tests and full reporting of outcomes
☒	☐	Estimates of effect sizes (e.g. Cohen's d, Pearson's r), indicating how they were calculated

Our web collection on statistics for biologists contains articles on many of the points above.

Software and code

Policy information about availability of computer code

Data collection	AlphaFold v2.3.2 was used to generate AlphaFold structure predictions.
Data analysis	Imaris File Converter v10.0.1, Imaris Stitcher v9.9.1, GraphPad Prism v10.0.2, Imaris v10.1, FlowJo 10.10.0, BWA-MEM v0.7.15, BBDuk v38.05, hisat2 v2.1.0, deepTools bamCoverage v3.0.1, Salmon v0.13.1, DESeq2 v1.38.3, ggplot2 v3.5.0, MACS2 v2.1.1, bedtools intersect v2.28.0, bedGraphToBigWig v4 ,SnapGene v7.0.1. and PAEViewer v1.0.0, Fiji v2.14.0/1.54f, Synthego ICE (https://ice.editco.bio/#/, v3.0), cistrome toolkit data browser (http://dbtoolkit.cistrome.org/), closestBed (v2.28.0), and the GREAT package v4.0.4. The mm10 genome was downloaded from https://genome.ucsc.edu/cgi-bin/hgGateway?db=mm10. Code used in this publication is available at https://github.com/adziulko/The-SP140-RESIST-pathway.

For manuscripts utilizing custom algorithms or software that are central to the research but not yet described in published literature, software must be made available to editors and reviewers. We strongly encourage code deposition in a community repository (e.g. GitHub). See the Nature Portfolio guidelines for submitting code & software for further information.

Data

Policy information about availability of data

All manuscripts must include a data availability statement. This statement should provide the following information, where applicable:

- Accession codes, unique identifiers, or web links for publicly available datasets
- A description of any restrictions on data availability
- For clinical datasets or third party data, please ensure that the statement adheres to our policy

HA-SP140 anti-HA CUT&RUN data is available at GEO accession: GSE269315. RNA-seq for for Ifnar–/– and Sp140–/–Ifnar–/– BMMs is available at GEO accession: GSE269761. RNA-seq and ATAC-seq for B6 and Sp140–/– BMMs is available at GEO accession GSE269808 and GSE269811 respectively.

Research involving human participants, their data, or biological material

Policy information about studies with human participants or human data. See also policy information about sex, gender (identity/presentation), and sexual orientation and race, ethnicity and racism.

Reporting on sex and gender	This information has not been collected.
Reporting on race, ethnicity, or other socially relevant groupings	This information has not been collected.
Population characteristics	This information has not been collected.
Recruitment	This information has not been collected.
Ethics oversight	This information has not been collected.

Note that full information on the approval of the study protocol must also be provided in the manuscript.

Field-specific reporting

Please select the one below that is the best fit for your research. If you are not sure, read the appropriate sections before making your selection.

☒ Life sciences ☐ Behavioural & social sciences ☐ Ecological, evolutionary & environmental sciences

For a reference copy of the document with all sections, see nature.com/documents/nr-reporting-summary-flat.pdf

Life sciences study design

All studies must disclose on these points even when the disclosure is negative.

Sample size	We did not pre-calculate sample sizes for our experiments. We generally chose sample sizes of at least 3 for in vitro experiments based on previous work (Ji and Witt et al, 2021) demonstrating the noise and variability in vitro macrophage experiments; these sample sizes, based on p values, were sufficient to show significant differences between groups. For in vivo experiments, we used sample sizes of 4-8 mice per group per independent experiment, based on previous observations of variability in groups for Legionella pneumophila in vivo infections (Ji and Witt et al, 2021).
Data exclusions	For RT-qPCR, data were excluded for predetermined criteria (bad ROX reference dye annotation or low housekeeping gene amounts indicative of RNA degradation).
Replication	All results were reproduced in at least 2 independent experiments.
Randomization	Samples were assigned based on mouse genotype, or randomized for assignment to treatment groups when possible. Covariates like sex and age were controlled for in vivo experiments and bone marrow-derived macrophage generation by matching these variables across genotype groups. Experiments were designed to equalize treatment conditions for all samples.
Blinding	Investigators were not blinded, as all results and analyses are based on objective data that was quantified by automated instruments or image analysis software.

Reporting for specific materials, systems and methods

We require information from authors about some types of materials, experimental systems and methods used in many studies. Here, indicate whether each material, system or method listed is relevant to your study. If you are not sure if a list item applies to your research, read the appropriate section before selecting a response.

Materials & experimental systems

n/a	Involved in the study
☐	☒ Antibodies
☐	☒ Eukaryotic cell lines
☒	☐ Palaeontology and archaeology
☐	☒ Animals and other organisms
☒	☐ Clinical data
☒	☐ Dual use research of concern
☒	☐ Plants

Methods

n/a	Involved in the study
☒	☐ ChIP-seq
☐	☒ Flow cytometry
☒	☐ MRI-based neuroimaging

Antibodies

Antibodies used	rabbit anti-HA monoclonal antibody (Cell Signaling Technologies, C29F4; 0.5 microgram per reaction), rabbit isotype control IgG (Epicypher, 13-0042; 0.5 microgram per reaction), rat anti-HA (Roche, clone 3F10, 1186742300; 1:1000 for WB, 1:200 for IF), mouse anti-actin (Santa Cruz Biotechnology, sc-47778; 1:1000), rabbit anti-CNOT1 (Cell Signaling Technologies, 44613S; 1:1000), rabbit anti-CNOT9 (Proteintech, 22503-1-AP; 1:500), rabbit anti-TTP (Millipore Sigma, ABE285; 1:1000), rabbit anti-CNOT11 (Sigma Aldrich, HPA069823; 0.4 microgram/mL), rabbit anti-ZFP36L1 (Cell Signaling Technologies, 30894S; 1:1000), rabbit anti-ZFP36L2 (Abcam, ab70775; 1:1000), rabbit anti FLAG (Thermo Fisher Scientific, PA1-984B; 1:1000) and rabbit-SP140 (Covance, as previously described in Ji and Witt et al 2021; 1:1000). Antibodies used in IF included the following: mouse anti-PML Millipore Sigma, 05-718, 1:100; rat anti-HA, Roche, 11867423001, 1:200; rabbit anti-Fibrillarin, Abcam, ab166630, 1:100. Secondary antibodies used in the study were donkey anti-rat Alexa Fluor 488, Invitrogen, A21208; goat anti-mouse Alexa Fluor 647, Invitrogen, A21236; goat anti rabbit 647, Life technologies, A21244. All secondary antibodies were used at a 1:1000 dilution.
Validation	Antibodies were validated by manufacturers based on statements on the manufacturer's websites (anti-actin: https://www.scbt.com/p/beta-actin-antibody-c4; anti-PML: https://www.sigmaaldrich.com/US/en/product/mm/05718; anti-fibrillarin: https://www.abcam.com/en-us/products/primary-antibodies/fibrillarin-antibody-epr10823b-nucleolar-marker-ab166630?srsltid=AfmBOorQJAsQoGYZO8WhIjr5ZQqUAFIjUy0ol8GN8bQFVCnb_zZDCHPz) and many antibodies within our publication were validated by inclusion of appropriate negative controls (for example, untagged samples were assessed for antibodies against epitope tags like HA). Anti-HA (Roche) was validated by the data included in Extended Data Figure 9b, d, and e, and Figure 5a; Anti-HA (CST) for CUT&RUN was validated by the inclusion of non HA-tagged SP140 controls (Extended Data Figure 1b). Anti-CNOT1, anti-CNOT11, and anti-CNOT9 were validated by data in Extended Data Figure 7a. Anti-TTP, anti-ZFP36L1 and anti-ZFP36L2 were validated by the data presented in Extended Data Figure 7a. Anti-FLAG (PA1-984B) was validated by the data present in Figure 3g. Anti-SP140 was validated by the data in Extended Data Figure 9b.

Eukaryotic cell lines

Policy information about cell lines and Sex and Gender in Research

Cell line source(s)	Cell lines (HEK293Ts and GP2 cells) were obtained from the UC Berkeley TC facility. BlaER1 cells originated from the lab of Veit Hornung. Bone-marrow derived macrophages from both male and female mice were used with no impact on obtained results.
Authentication	All cell lines were authenticated by STR profiling.
Mycoplasma contamination	Cell lines were tested by PCR for mycoplasma contamination, and were not positive.
Commonly misidentified lines (See ICLAC register)	No commonly misidentified lines were used in this study.

Animals and other research organisms

Policy information about studies involving animals; ARRIVE guidelines recommended for reporting animal research, and Sex and Gender in Research

Laboratory animals	This study involved the laboratory animal Mus musculus, strain C57BL6/J, and strain B6.129S2-Ifnar1tm1Agt/Mmjax, and Sp140–/– mice generated in Ji and Witt et al, Elife, 2021 on the C57BL6/J background. Sp140–/–Resist1/2–/– mice were also generated on the C57BL6/J strain background for this publication as described in publication methods. Mice were used for derivation of bone-marrow derived macrophages between 6-26 weeks of age, and for infection with Legionella pneumophila between 8 and 30 weeks of age.
Wild animals	The study did not involve wild animals.
Reporting on sex	Male and female mice were used for bone-marrow derived macrophage differentiation and for Legionella pneumophila infections, with no observable difference in obtained results based on sex. Within bone marrow macrophage derivation experiments, mice of one sex were matched across genotypes.
Field-collected samples	The study did not involve collection of field-samples.

Ethics oversight	All experiments were performed following the University of California Berkeley Institutional Animal Care and Use Committee regulatory standards.

Note that full information on the approval of the study protocol must also be provided in the manuscript.

Plants

Seed stocks	Not applicable
Novel plant genotypes	Not applicable
Authentication	Not applicable

Flow Cytometry

Plots

Confirm that:

- [x] The axis labels state the marker and fluorochrome used (e.g. CD4-FITC).
- [x] The axis scales are clearly visible. Include numbers along axes only for bottom left plot of group (a 'group' is an analysis of identical markers).
- [x] All plots are contour plots with outliers or pseudocolor plots.
- [x] A numerical value for number of cells or percentage (with statistics) is provided.

Methodology

Sample preparation	BMMs infected with MHV68-GFP were harvested in PBS, stained with Ghost Dye Far Red 780, fixed with the BD Cytofix Cytoperm kit according to kit instructions, then washed and analyzed by flow cytometry.
Instrument	BD LSR Fortessa X-20 and BD LSR Fortessa were used to generate flow cytometry data in this study.
Software	FlowJo version 10 (BD Biosciences)
Cell population abundance	No samples in this study were sorted.
Gating strategy	Samples were gated on SSC and FSC, then gated on single cells based on FSC-W and FSC-A, then gated on live cells based on Far-Red 780 staining. MHV-68 GFP positive gates were drawn based on uninfected controls.

- [x] Tick this box to confirm that a figure exemplifying the gating strategy is provided in the Supplementary Information.
